# The Factors that Induce or Overcome Freezing of Gait in Parkinson’s Disease

**DOI:** 10.1155/2008/456298

**Published:** 2008-07-15

**Authors:** S. Rahman, H. J. Griffin, N. P. Quinn, M. Jahanshahi

**Affiliations:** Sobell Department of Motor Neuroscience and Movement DisordersInstitute of Neurology and the National Hospital for Neurology and NeurosurgeryUniversity College LondonQueen SquareLondonWC1N 3BGUK

**Keywords:** Parkinson’s disease, freezing, gait, emotional modulation, external cueing, paradoxical kinesis, mobility

## Abstract

Freezing of gait (FoG), a transient halt in walking, is a major mobility problem for patients with Parkinson’s disease (PD). This study examined the factors that induce FoG, and identified the cues and strategies that help overcome it through a postal survey of 130 PD patients. 72% reported FoG. The factors that commonly induced FoG were turning, fatigue, confined spaces and stressful situations, in addition to emotional factors. FoG was also ameliorated by various attentional and external cueing strategies. The concept of paradoxical kinesis, the potential neural substrates of such external cueing effects, and their importance for rehabilitation in PD are discussed.

*Abbreviations:* FoG – freezing of gait, H&Y – Hoehn and Yahr, PD – Parkinson's disease, QoL – quality of life

## 1. Introduction

In idiopathic Parkinson's disease (PD), freezing of gait (FoG) is a mobility problem where walking becomes hesitant and may cease entirely. This is described by patients as if their feet are 'glued to the floor' [1,2]. This type of motor block, can be accompanied by postural instability and subsequent falls [3, 4], and can be unresponsive to treatment, either medication [5] or surgery [6,7]. Therefore, the management of mobility problems in PD could benefit from a multidisciplinary approach, informed by an understanding of the factors that induce or help to overcome FoG, and of other specific mobility problems such as gait initiation failure or shuffling. A greater understanding of these factors in PD may also be crucial to management of FoG in other parkinsonian syndromes [8].

Past studies have shown that FoG episodes occur when the patient encounters certain obstacles or meets a constriction in visual or proprioceptive input. Situations in which FoG is likely include turning, starting to walk, narrow spaces such as doorways or lavatories, stressful, rushed situations such as hearing a knock at the door or when an elevator door opens, and approaching a destination [1,8–10]. As PD progresses, FoG may appear even in open spaces in the absence of any external trigger, highlighting its unpredictability. These survey-based findings have been difficult to corroborate in controlled conditions, since FoG is episodic and tends not to occur during visits to the clinic or laboratory. However, Schaafsma et al. [11] elucidated the influence of potential FoG "triggers" by having PD patients known to suffer from FoG perform a continuous mobility task incorporating several such items. Turning most reliably elicited FoG with starting, walking through a narrow gap, and approaching a destination eliciting FoG in a smaller percentage of trials. FoG has been found not to be associated with bradykinesia and rigidity [12,13], suggesting that it is distinct from other PD symptoms that restrict mobility.

Conversely, certain situations, cues or strategies improve mobility in PD [9]. Luria [14], and Souques [15], gave examples of how patients with PD could run up-

ISSN 0953-4180/08/$17.00 © 2008 – IOS Press and the authors. All rights reserved stairs but seemed unable to walk on an even surface. Purdon-Martin [16] documented the facilitative effects of visual cues on Parkinsonian gait, noting a marked improvement, particularly in stride length, when a patient stepped across lines placed transversely at appropriate intervals on the floor. This phenomenon was considered to explain why many patients with PD are able to mount stairs better than they can walk on a level surface, with each stair acting as an external cue. Since this observation a number of investigations have reported the effects of specifically providing visual, auditory or tactile cues as a mobility aid [17–24], for review see [25] and noted differences between PD patients suffering from freezing and those who do not [26].

While there is an overall consensus that FoG does not occur randomly, and that sensory cues can both trigger and ameliorate FoG in PD; the relative contributions of the modality of sensory cues to this phenomenon, and of other more internally-generated modulation such as by attention or emotional stimuli, remain unclear. The main purpose of the current study was (i) to obtain information about the factors that induce FoG in PD whether externally (sensory cues) or internally (attention, emotion) triggered, and (ii) to assess which strategies or external cues are successful in overcoming FoG or improving walking in PD.

## 2. Methods

### 2.1. Recruitment

This study was approved by our local Ethics Committee. Informed consent was obtained from all participants. Participants were recruited from two sources: (i) we invited patients who had recently attended PD clinics at the NHNN to participate, (ii) we advertised in the newsletter of the Parkinson's Disease Society (PDS). The advert invited patients to take part in a "mobility survey", but did not refer to freezing. 60 patients replied to the advertisement in the PDS newsletter; 68% of the recent NHNN patients invited also completed the questionnaire, giving a patient cohort of 130 patients (84 male) in total. The mean age of the patients was 66.7 (SD 8.52). The mean duration of illness was 12.1 (SD 7.94). Patients classified their stage of illness on the revised Hoehn & Yahr scale [27], which provided short descriptions of the main features of the illness at each stage. In a population-based study of depression and quality of life in PD, patient self-ratings and neurologist ratings of stage of illness were obtained and shown to be strongly correlated [28]. Mean self-rated Hoehn & Yahr (H&Y) stage [27] was 2.63 (SD = 1.19). The majority of questions could be answered by marking the chosen response and no writing was required.

### 2.2. Freezing of gait

FoG was assessed using the gait and falls questionnaire (GFQ) [2]. 16 items detailing the frequency and severity of freezing, falling and festination were rated on a scale of 0 (no impairment) to 4 (most severe impairment). Freezing was explained in question 4: "Do you feel that your feet get glued to the floor while walking, making a turn or when trying to initiate walking (freezing)?" Patients who responded $\geqslant 1$, i.e., that they experienced this "about once a month" or more often were defined as "freezers". Falling as a result of FoG was assessed in question 14: "How often do you fall because of freezing episodes?"

### 2.3. Factors influencing walking/freezing

Patients indicated whether specific factors (fully listed in Table 2) induced freezing for them. They also noted whether visual, auditory and tactile stimuli or emotional factors improved their mobility and whether specific external stimuli or certain "trick" strategies (fully listed in Table 4) influenced their walking or freezing by marking: 0 – not applicable, 1 – mild improvement, 2 – moderate improvement, 3 – marked improvement. An open-ended question was also asked about whether emotional factors either induced or improved FoG.

### 2.4. Statistics

The proportion of patients experiencing freezing is reported as a percentage of the total cohort. The differences in proportion with FoG between groups (H&Y stage, sex) were assessed using chi-square ($X^2$) tests, with Yates correction applied where $df = 1$; 1-tailed p-values were calculated only where a clear prediction existed. The numbers of patients who reported that a specific item triggered or overcame freezing are reported as percentages of the entire cohort alongside the number of responders (valid N) for each item in Tables 2 and 4.

## 3. Results

### 3.1. Freezing of gait (FoG)

The responses to the freezing-related items of the GFQ are detailed in Table 1. Most importantly, 72.3% of patients had suffered from freezing; with 46.2% experiencing it at least daily. All freezing triggers typically induced freezing episodes lasting 3–10s, although for spontaneous freezing a shorter episode of 1–2s was equally likely. 30.0% of patients had experienced a fall secondary to a freezing episode.

There was no difference between the proportion of male and female patients reporting FoG (72.6% and 71.7% respectively; $X^2 = 0.011$, $df = 1$, $p = 0.9$, 2-tailed). The number of non-freezers was too small to allow $X^2$ comparisons of all H&Y stages with each other. Patients were therefore allocated to 3 groups: *mild* (H&Y stages 0–1.5, $N = 29$), *moderate* (H&Y stages 2–3, $N = 59$) and *severe* (H&Y stages 4–5, $N = 38$), with 4 patients omitted because they did not submit a self-rated H&Y stage. The proportion of freezers was contingent on the H&Y stage ($X^2 = 10.0$, $df = 2$, $p = 0.007$, 2-tailed) with 51.7% of the mild, 83.1% of the moderate, and 76.3% of the severe group reporting FoG. Pairwise comparisons revealed, as expected, greater freezing in the moderate ($X^2 = 8.10$, $df = 1$, $p = 0.003$, 1-tailed) and severe ($X^2 = 3.39$, $df = 1$, $p = 0.04$, 1-tailed) groups than the mild group, but no significant difference between the moderate and severe groups ($X^2 = 0.307$, $df = 1$, $p = 0.2$, 2-tailed). Freezers (mean age = 66.4, $SD = 8.20$) were no older than non-freezers (mean age = 67.2, $SD = 9.82$; F(1,113) = 0.176, $p = 0.6$).

### 3.2. The factors that induce freezing

Table 2 shows the percentage of patients who reported that each of the specific triggers induced freezing, and the number of responders (valid N) for each item. The mean valid N was 101. Turning around, fatigue, being in a confined space and stressful situations were freezing 'triggers' for more than half of patients. Narrow spaces or crowds, going through doorways, getting out of a chair, being surrounded by people who are talking, panic attacks, crossing roads and entering a lift as the door opens were reported as causing freezing by 30 to 49% of responders. Additionally 35.4% of patients reported that emotional factors, such as anxiety, fear or depression, affected their freezing. These results are consistent with the data from the GFQ (Table 1) in which the largest percentage of patients reported hesitation from turning and the smallest percentage reported spontaneous freezing while walking on open ground.

Table 3 shows the 6 most problematic triggers in each group with the percentage of patients from each stage who reported them as eliciting freezing. The relative importance of these trigger across PD stages is fairly consistent: turning and fatigue are among the 3 most common triggers in all stages. The relative importance of being in a stressful situation, being in a crowd or confined space, and walking in a narrow space is more variable. In addition, some triggers that are relatively inconsequential in early stage PD become important in later stages: "Being surrounded by people talking", increases from the 21st most important trigger (6.9% of patients in the mild group) to the 7th most important (57.9% of patients in the severe group); and "Crossing roads", increases from the 18thmost important trigger (10.3% of patients in the mild group) to the 9th most important (52.6% of patients in the severe group).

### 3.3. The factors that improve freezing or walking

The percentage of the sample who reported that cues in particular modalities improved their mobility were: visual 31.5%, tactile 27.7%, emotional 26.2%, auditory 19.2%. Table 4 shows the percentage of patients who reported that specific strategies improved their walking or freezing and the number of responders (valid N) for each item, mean valid N was 104. Paying attention to every step was the only strategy that was reported as having improved their walking or freezing by more than half of patients. Taking longer steps, altering the distribution of body weight, going up stairs, consciously lifting one limb higher than another, stamping feet and counting silently were reported by 30 to 49% of responders as improving walking or freezing.

## 4. Discussion

The high percentage of patients, 72.3%, suffering from FoG supports previous findings that freezing is common. The higher rate of FoG in our sample compared to previous studies [1,5,10] may be due to several factors including different methods of assessing FoG. While a clear definition of FoG was provided, it is nevertheless possible that therapy-related fluctuations such as end of dose wearing off may have been confused with freezing by a minority of patients. Our sample

Table 1
Responses to gait and falls questionnaire. Frequency of freezing and length of freezing episodes. Percentages are of entire cohort

| | **Never** | **About once a month** | **About once a week** | **About once a day** | **Whenever walking** | **No response** |
|---|---|---|---|---|---|---|
| Q4: Do you feel that your feet get glued to the floor. . . (freezing)? | 19.2% | 10.0% | 16.2% | 33.1% | 13.1% | 8.5% |
| | **Never happened** | **1–2 s** | **3–10 s** | **11–30 s** | **>30 s** | **No response** |
| Q5: Longest freezing episode | 21.5% | 16.9% | 26.9% | 11.5% | 13.8% | 9.2% |
| | **None** | **>1 s** | **>3 s** | **>10 s** | **>30 s** | **No response** |
| Q6: Typical start hesitation episode | 20.0% | 22.3% | 26.9% | 13.8% | 6.9% | 10.0% |
| Length of typical. . . | **None** | **1–2 s** | **3–10 s** | **11–30 s** | **>30 s** | **No response** |
| Q7: Turning hesitation | 26.2% | 20.0% | 29.2% | 7.7% | 7.7% | 9.2% |
| Q8: Destination hesitation | 35.4% | 19.2% | 23.8% | 7.7% | 4.6% | 9.2% |
| Q9: Tight quarters hesitation | 35.4% | 15.4% | 23.1% | 9.2% | 6.2% | 10.8% |
| Q10: Freezing while walking on straight | 46.9% | 16.2% | 16.2% | 5.4% | 5.4% | 10.0% |
| Q11: Freezing during stressful, time-demanding situations | 32.3% | 14.6% | 27.7% | 6.9% | 8.5% | 10.0% |
| | **Never** | **Once or twice** | **3–12 times in last 6 months** | **> once per week** | **Whenever walking unassisted** | **No response** |
| Q14: Falling because of freezing? | 60.8% | 16.9% | 6.9% | 5.4% | 0.8% | 9.2% |

Table 2
Factors which induced freezing of gait

| Factor | % yes of sample | Number of responders (valid N) |
|---|---|---|
| Turning around | 58.5% | 107 |
| Fatigue | 58.5% | 107 |
| Being in a confined space | 53.1% | 103 |
| Being in a stressful situation such as approaching or entering a crowd | 53.1% | 105 |
| Walking in a narrow space such as a narrow corridor or between objects | 49.2% | 105 |
| Being in a crowd | 49.2% | 105 |
| Going through doorways | 43.8% | 103 |
| Getting out of a chair | 40.0% | 104 |
| Being surrounded by people who are talking | 35.4% | 102 |
| Panic attacks | 32.3% | 104 |
| Crossing roads | 30.8% | 97 |
| Entering a lift as the door opens | 30.0% | 99 |
| Hearing a doorbell or telephone ring | 29.2% | 103 |
| Stepping onto an escalator | 25.4% | 97 |
| Reaching for doorhandles | 24.6% | 100 |
| Approaching a target or a destination | 24.6% | 100 |
| Meeting somebody unexpectedly | 23.8% | 105 |
| Getting off a bus | 23.1% | 86 |
| Getting on a bus | 22.3% | 87 |
| Using cutlery such as putting a fork or spoon into your mouth | 18.5% | 103 |
| Loud noise | 17.7% | 102 |
| Using a computer mouse | 16.2% | 93 |
| Bright lights | 11.5% | 99 |

was recruited from a specialist Movement Disorders Clinic and through an advertisement to take part in a "mobility survey", therefore the responders may disproportionately have been PD patients with mobility problems. However, no mention was made of freezing in the advert, and there is no reason to believe that there was a selection bias in this respect. Therefore, while our methods of recruitment may have led to over-representation of PD patients with mobility problems in our sample, the FoG figures are unlikely to be over-inflated. Indeed, from a review of the literature, it has been suggested [4] that the proportion of PD patients experiencing FoG is likely to be higher than the 20–60% rates previously reported, partly due to its episod-

Table 3
The most commonly reported freezing triggers in different stages of PD. Percentages are of all patients in group

| | Mild (H&Y stage 0–1.5) | Moderate (H&Y stage 2–3) | Severe (H&Y stage 4–5) |
|---|---|---|---|
| Most common | Turning (48.3%) | Stressful situations (54.2%) | Fatigue & Walking in a narrow space (76.3%) |
| 2nd | Fatigue (44.8%) | Fatigue & Turning (52.5%) | |
| 3rd | Confined spaces (41.4%) | | Turning (73.7%) |
| 4th | Doorways (34.5%) | Confined spaces & Being in a crowd (47.5%) | Stressful situations & Confined spaces (68.4%) |
| 5th | Getting out of a chair (31.0%) | | |
| 6th | Stressful situations & Walking in a narrow space & Being in a crowd (27.6%) | Walking in a narrow space & Getting out of a chair (40.7%) | Being in a crowd (65.8%) |

ic nature. More than one quarter of the patients in our study had experienced falls secondary to freezing. Thus these mobility problems are likely to have a large 'cost' for people with PD in terms of fractures, confinement, inducing fear of falling, social isolation, and poor quality of life (QoL) [4,29,30].

### 4.1. Factors that induced freezing

Most striking from the patients' responses is that endogenous triggers such as fatigue and stress are among the most common freezing triggers, reported by more than half of patients, alongside specific situations such as turning or confined spaces. Like all aspects of PD, the management of freezing should be tailored to the specific needs of the individual patient. However, these results do identify the most common freezing triggers such as fatigue and turning, which should be targeted for further general research in order to maximize benefit to patients with FoG at all stages of the disease. Additionally, some triggers, such as being surrounded by people who are talking and crossing roads, are reported by relatively few patients in the early stages of PD, but are among the most common triggers in later stages. The responses to this survey cannot reveal precisely what aspects of these situations cause freezing, but it is noteworthy that they are dynamic situations with many distractions compared to, for example, being in a confined space. These triggers might therefore best be addressed using suitable solutions e.g., attentional strategies, specifically developed with the symptoms and limitations of late-stage PD patients e.g., impaired balance, in mind.

Whilst stress can undoubtedly improve mobility in specific situations, such as paradoxical kinesis induced by a fire, our results also highlight the significance of stress as a *trigger* for freezing. One patient remarked about the effects of stress on freezing, "*... mostly if I am running late for something, it can be appointments or cooking a meal and any form of stress*". The example of a stressful situation given in the questionnaire is "approaching or entering a crowd"; problematic crowded places for PD patients include supermarkets, railway stations and town centres. These situations are likely to have a number of properties that make their navigation difficult for people with PD, particularly in light of a possible distortion of representations of external space in PD [31], and the fact that crowds themselves create confined spaces. A specific association between panic and freezing was apparent as in previous studies [32]. One patient stated, "*I get panic attacks during journeys. Awareness of previous panic attacks and thinking through the problems before the activity affect freezing*". Another patient noted: "*freezing and panic attacks came together as a package for me. One triggered the other, it became a vicious circle, extremely difficult to overcome*". The substantial anxiety component that these comments illustrate may even produce agoraphobia, an intense fear of feeling trapped and having a panic attack in a public space, which has been reported alongside other anxiety disorders including social anxiety in PD patients [33]. Cognitive-behavioural therapy may help patients overcome some of these fears and concerns, and manage stress in order to reduce freezing episodes.

The interplay between emotion and freezing may have an underlying biological basis in the rich functional interconnectivity between prefrontal and limbic regions of the cerebral cortex. Using SPECT, Matsui and colleagues [34] found that bilateral BA11 perfusion (i.e. the ventromedial prefrontal cortex) was decreased in PD patients with FoG compared to those

Table 4
Strategies which helped overcome freezing or improve walking

| Strategy | % yes of sample | Number of responders (valid N) |
|---|---|---|
| Paying attention to every step | 53.8% | 107 |
| Taking longer steps | 47.7% | 108 |
| Altering the distribution of body weight | 44.6% | 107 |
| Going up stairs | 43.1% | 104 |
| Consciously lifting one limb higher | 35.4% | 104 |
| Stamping feet | 32.3% | 104 |
| Counting silently | 31.5% | 107 |
| Walking sideways crablike | 28.5% | 104 |
| Humming a military march or marching to command like a soldier | 28.5% | 108 |
| Following lines on the floor | 24.6% | 103 |
| Saying something provocative to yourself | 24.6% | 103 |
| Counting aloud | 23.8% | 103 |
| Visualising beyond an obstacle such as a doorway | 23.1% | 104 |
| Changing direction | 22.3% | 103 |
| Walking over the edges of tiles/paving stones | 20.8% | 103 |
| Clapping of hands | 18.5% | 102 |
| Another person giving verbal commands | 18.5% | 104 |
| Singing a song | 18.5% | 105 |
| Stepping over an upturned walking stick | 16.2% | 103 |
| Calling for help | 15.4% | 101 |
| Watching other people walk | 14.6% | 104 |
| Stepping over a carer's foot | 13.8% | 100 |
| Walking down a street and counting the house numbers | 10.0% | 103 |
| Walking to a metronome | 8.5% | 102 |
| A piece of paper placed in front of your feet | 6.9% | 103 |
| Using masking tape on the floor | 6.2% | 101 |
| Sudden clapping of hands by another person | 6.2% | 104 |
| Imagining white lines on a highway | 6.2% | 106 |
| Sudden jerky head movements | 3.8% | 99 |
| Walking over pretend letters of the alphabet | 3.8% | 104 |

without. The distributed neuronal network involving the orbitofrontal cortex [BA 11, 13 and 47/12] also receives input from all the sensory modalities, including visceral afferents (for review see Kringelbach [35]). This may represent a locus for the interaction between emotion and movement. On a more symptomatic level, in a review of the literature, Macht and Ellgring (1999) [36] proposed a behavioural model of freezing, whereby external factors, for example limited time, confined space or stress led to reactions during the freezing episode such as tension, fear, negative cognitions, anger, in turn with consequences of despair and helplessness. In their proposed model, anxiety, fear and stress can both cause and be consequences of freezing.

The reported effect of fatigue as a trigger of FoG is in agreement with previous findings, and fatigue has been noted to impair patients' utilisation of cues as a mobility aid [22]. Fatigue is a disabling PD symptom [37], which has a significant impact on QoL of patients [29]. Given the importance of fatigue to both FoG and QoL, its direct management would be of value for PD patients.

In terms of specific actions, difficulty in turning was the most common freezing trigger, in agreement with previous studies [11]. Difficulty turning is characteristic of PD [38], and is associated with falls [39] as well as poorer QoL [29]. PD patients are known to take more steps to change direction [39]. High-frequency stepping and a wide base of support may be a cause of and/or compensation for difficulty in turning and may indicate the sacrifice of efficacy for stability [40]. Given the association between difficulty in turning, freezing and falls, patients need a clear home environment to reduce the need to turn and to lessen the risk of injury on landing [39].

Getting out of a chair was a further difficulty experienced as inducing freezing by large percentage of the sample. This activity requires a complicated sequencing of muscle activity. PD patients are often advised to rehearse a sequence of movements and rely on a series of subvocal commands to overcome the problem, such as 'move to the edge of the chair, hands on chair arms, feet flat on floor, nose over toes, head up, stand tall.' [41]. We found that around 44% of patients reported that going through doorways caused freezing. The traditional explanation for this has been that edges of doorways, or other objects whose images fall in the peripheral field of vision, may somehow capture attention, such that a PD patient is unable to devote enough resources to controlling voluntary movement and hence freezes [42], consistent with a hypothesis that abnormal visual processing interferes with motor initiation [43]. Problems with entering lifts and crossing roads were problematic for more than 30% of patients and potentially disabling in an urban environment. Similarly entering or exiting a bus is a freezing trigger for more than one patient in five. Strategies for overcoming such problems would be valuable for those patients for whom they are a part of everyday life.

### 4.2. *Factors that overcome freezing episodes or improve walking*

Paying attention to every step, taking longer steps, altering the distribution of body weight, and going up stairs were reported by $>$40% of the sample to improve walking and overcome FoG. With the exception of going up stairs, the strategies most commonly reported as effective required no external objects/cues. Rochester and colleagues [22,44] conceptualize such thinking about the movement or the size of a step, as a 'cognitive-attentional cueing' strategy, in keeping with Morris et al. [45] who demonstrated improvements in stride length after the use of visual or attentional cues, when conducting a secondary task while walking. Altering distribution of body weight is recommended by physiotherapists who advise patients not to try to move forward when they freeze, but instead to shift gently the majority of their weight sideways to one leg [46].

Visual cues were reported to be useful by more than 30% of the sample. The most commonly helpful visual cues, following lines on the floor or walking over the edges of tiles/paving stones were consistent with the findings of Purdon-Martin [16]. Other visual strategies reported as less commonly helpful have also appeared in the literature. Consistent with our results, Dietz et al. [47] reported that only a small subpopulation of PD patients benefited from use of a modified walking stick. Dunne, Hankey and Edis [48] reported three parkinsonian patients who felt that their gait had been facilitated by inverting a walking stick and using the handle, especially in relation to a difficulty in step initiation and maintenance of stride length. The idea is that the visual cues focus the patients' attention, allowing reliance on alternative, more conscious motor control pathways in the regulation of gait. The benefit of walking up stairs may be that it invokes such visual and attentional mechanisms. Azulay et al. [23] hypothesised that visual cues serve as moving targets, activating a cerebellar visual-motor pathway. Such beneficial effects of visual cues on gait in PD would therefore have implications for the development of gait facilitation devices such as virtual reality glasses which could, for example, use moving steps as a useful stimulus.

Auditory cues have been tested as strategies for improving gait in PD e.g. [17–22] and been found effective in some [18], but not other studies [17]. Here, they are reported as the least useful modality of cue. There is evidence that auditory cues may invoke beneficial changes in neural plasticity from a study involving a four week programme with one hour daily training with rhythmic auditory cues, which appeared to produce improvements in gait in patients with PD [49]. PET analysis demonstrated an increased glucose uptake in the right anterior lobule of the cerebellum and dentate nucleus near the midline and in the right temporo-parietal conjunction after therapy. This increase, together with the improvement in performance of a motor task by their patients after therapy, suggested a switch in neural function to alternative cerebellar pathways to compensate for the dysfunctional putaminal-SMA/prefrontal circuits.

A recent international study is the most comprehensive investigation of the efficacy of visual, auditory and tactile cueing [24]. PD patients were trained with rhythmic cueing in the modality of their choice in nine, thirty-minute sessions over three weeks. Before and after this training the patients were tested in the absence of the cues in order to assess the carry-over effects of such training. The training resulted in small, specific improvements in gait. Freezers did show an improvement on the FoG questionnaire [2] of 5.5%. The mobility benefits seen at the end of the training were not sustained at a 6-week follow up, a result that the authors attribute to the relatively sparse nature of the training.

### 4.3. *Limitations of this study*

The advantage of large postal surveys such as this is that the symptoms of a large number of patients can be assessed simultaneously. One obvious disadvantage is that the reports of the patients and their staging of their illness are not confirmed by assessment by a clinician. However, with regard to freezing, this methodological problem can not be easily overcome by direct assessment since freezing is an episodic and sporadic phenomenon and may not occur during meetings between patient and clinician [4]. Furthermore, information about the range of factors that induced or resolved freezing episodes in real life situations can only be obtained from the patients themselves.

The recruitment of patients to take part in a "mobility survey" may have led to an over-representation of patients with mobility issues, in comparison to the wider population of Parkinson's patients. However, there is no reason to believe that the 72.3% is an overestimation of the percentage of patients with mobility problems who experience freezing. Recall bias is a further complication that must be considered. Arguably patients are more likely to recall triggers that are more consistent in producing freezing, or those that elicit more prolonged, embarrassing or functionally inconveniencing freezing episodes. It is possible therefore that items reported as freezing triggers by a relatively small proportion of patients are actually under-represented. However, the agreement with previous studies [8–11] of the relative importance of particular freezing triggers is encouraging. One possible solution is a "freezing diary" in which patients record all freezing episodes, and the associated situation that triggered or helped resolve the freezing episode, shortly after they occur. Problems with motivation and consistency of recording would have to be overcome, but this approach has potential for indicating what practical, real-world solutions for freezing exist.

### *4.4. Conclusions*

Our results on the reports of the positive effects of external cueing on mobility are of particular interest in relation to the concept of 'paradoxical kinesis', which originally referred to the phenomenon of sudden mobility in akinetic PD patients induced by *emotional* stimuli such as a fire or the sight of a drowning man. MacDonald Critchley, in a classic manuscript on the nature of arteriosclerotic Parkinsonism published in *Brain* in 1929 [50], noted: "The influence of the emotions upon these more automatic movements is frequently striking; under the influence of sudden fear or during an instinctive response to danger, the gait may lose its feeble and short-stepping characters and approximate to the normal." Paradoxical kinesis undoubtedly has a neurobiological basis [51]. For example, it is possible that in PD potent emotional stimuli result in a release of endogenous dopamine which mediates the temporary improvement in mobility, a hypothesis worth investigating in future studies. Alternatively, paradoxical kinesis may represent improved mobility mediated by an interaction between motor and limbic fronto-striatal circuits. The current results provide important information about the factors that induce freezing or ameliorate gait, which may in the future prove of value in the development of new rehabilitative technologies such as virtual reality mobility aids that utilize the power of external stimulation for PD patients.

## Acknowledgements

We are grateful to all patients who volunteered to participate in this study. This work was funded by the UK Parkinson's Disease Society.
